# Association between *TP53* Arg72Pro polymorphism and leukemia risk: a meta-analysis of 14 case-control studies

**DOI:** 10.1038/srep24097

**Published:** 2016-04-07

**Authors:** Xin Tian, Shundong Dai, Jing Sun, Shenyi Jiang, Youhong Jiang

**Affiliations:** 1 Molecular Oncology Laboratory of Cancer Research Institute, the First Affiliated Hospital of China Medical University, Shenyang, 110001, P.R. China; 2 Department of Pathology, the First Affiliated Hospital and College of Basic Medical Sciences of China Medical University, Shenyang, 110001, P.R. China; 3 Institute of Pathology and Pathophysiology, Shenyang, 110001, P.R. China; 4 Department of Immunology and Biotherapy, Liaoning Cancer Hospital and Institute, Shenyang, 110042, P.R. China; 5 Department of Rheumatology, the First Affiliated Hospital of China Medical University, Shenyang, 110001, P.R. China

## Abstract

The relationship between the *TP53* Arg72Pro polymorphism (rs1042522) and the risk of leukemia remains controversial. Consequently, we performed a meta-analysis to accurately evaluate the association between *TP53* Arg72Pro polymorphism and leukemia risk. A comprehensive search was conducted to find all eligible studies of *TP53* Arg72Pro polymorphism and leukemia risk. Fourteen case-control studies, with 2,506 cases and 4,386 controls, were selected for analysis. The overall data failed to indicate a significant association between *TP53* Arg72Pro polymorphism and the risk of leukemia (C vs. G: OR = 1.09, 95% CI = 0.93–1.26; CC vs. GC + GG: OR = 1.23, 95% CI = 0.96–1.57). In a subgroup analysis of clinical types, an increased risk was observed in the acute lymphocytic leukemia (ALL) subgroup (CC vs. GC + GG: OR = 1.73; 95% CI = 1.07–2.81) but not in the acute myeloid leukemia (AML) subgroup. In the subgroup analysis, no significant associations with ethnicity and the source of the controls were observed. In conclusion, the results suggest that there is no association between *TP53* Arg72Pro polymorphism and the risk of leukemia, but the CC genotype may increase the risk of ALL *TP53* Arg72Pro polymorphism CC genotype may increase the risk of ALL but is not associated with AML. Further large-scale, well-designed studies are needed to confirm our results.

Leukemia is a malignant tumor of the hematopoietic systems. It has a poor prognosis due to a range of complicated features^1^. Based on the speed of onset and cytogenetic analysis, leukemia was divided into four main types^2^: acute lymphocytic leukemia (ALL), chronic lymphocytic leukemia (CLL), acute myeloid leukemia (AML), and chronic myeloid leukemia (CML). Although leukemogenesis studies have been conducted for many years, the mechanisms underlying the development of this hematologic malignancy remain unclear. Multiple risk factors are thought to be implicated in leukemia, and genetic factors, such as constitutional genetic variation in components of DNA damage response pathways, have become the focus of research^3^,^4^.

The TP53 tumor suppressor gene, which is located on chromosome 17p13, plays a pivotal role in the maintenance of genomic stability through encoding of the TP53 protein. The TP53 protein functions by regulating cell cycle arrest, DNA repair, apoptosis, and gene transcription to mediate cellular responses to DNA damage^5^. Frequent mutations and differential expression of TP53 in various cancers highlight the significance role of p53 in carcinogenesis and tumor progression^6^,^7^. A functional single-nucleotide polymorphism (SNP) at codon 72 of TP53 gene (rs1042522), encoding a transversion of G to C (Arg to Pro), has been demonstrated to be associated with interindividual differences of TP53 expression to malignant tumors, including leukemia^8^. In addition, it has been reported that the Arg72 variant induces apoptosis markedly better compared to the Pro72 variant^9^. Thus, this genetic polymorphism holds promise as a potential biomarker for leukemia.

To date, numerous studies have investigated the relationship between the *TP53* Arg72Pro polymorphism and predisposition to leukemia, but the impact of *TP53* Arg72Pro polymorphism on leukemia was still conflicting due to inconsistent findings in individual studies. It was necessary to quantitatively summarize the evidence using the gradually accumulated data. Therefore, the current meta-analysis of the 14 most recent and relevant case-control studies involving 2,506 cases and 4,386 controls was performed to provide a more precise estimate of the associations.

## Results

### Characteristics of studies

**Figure 1:**
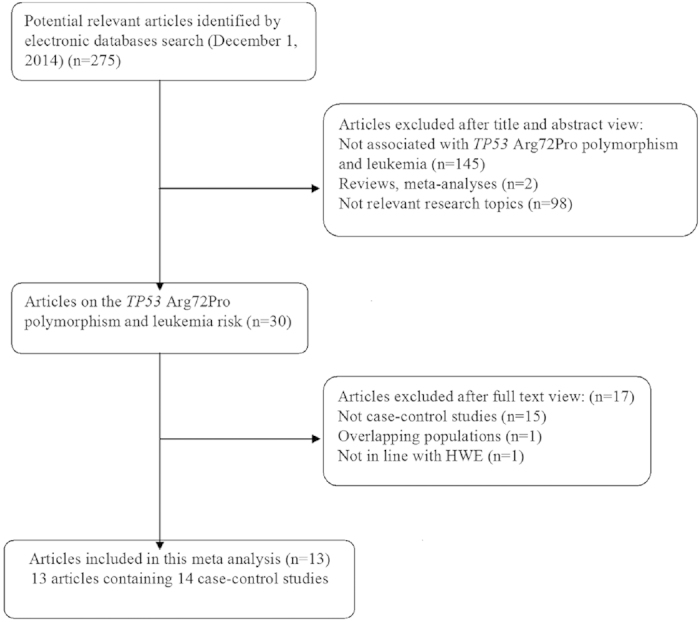
Flow chart of publication selection procedure.

A total of 14 case-control studies that examined the association of *TP53* Arg72Pro polymorphism with leukemia were included in the meta-analysis^10^,^11^,^12^,^13^,^14^,^15^,^16^,^17^,^18^,^19^,^20^,^21^,^22^. As shown in the flowchart of the selection process of the included studies (Fig. 1), one article was excluded because it was conducted on overlapping populations with another eligible study^23^; one article was excluded because the gene distribution of control group deviated from the HWE^24^; and one article involved two independent case-control studies that were considered separately, resulting in two studies^14^. Of the 14 studies, 8 were conducted in Asian populations, 5 in Caucasian populations, and one in a mixed population. There were 7 AML studies and 5 ALL studies, the remaining studies were CML, CLL, ATL and mixed types. There were 12 population-based studies and 2 hospital-based studies. The main characteristics of the selected studies and the genotype distribution of the *TP53* Arg72Pro polymorphism are summarized in Table 1.

### Meta-analysis Results

**Figure 2:**
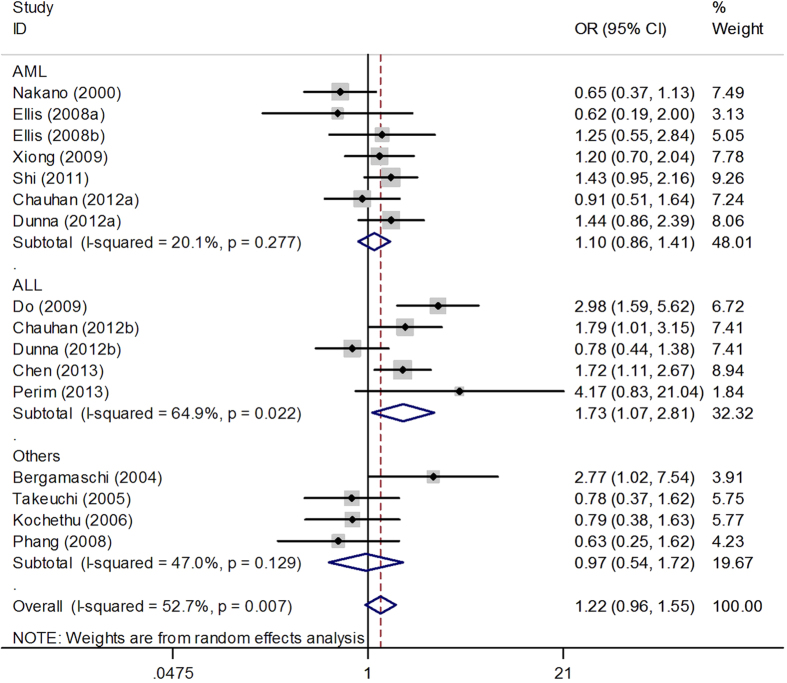
Forest plot of OR with 95% CI of leukemia associated with the *TP53* Arg72Pro polymorphism according to the clinical type group (recessive model).

Table 2 lists the main results of this meta-analysis. The results of pooling all studies showed that there was no statistically significant association between *TP53* Arg72Pro polymorphism and the risk of leukemia (C vs. G: OR = 1.09, 95% CI = 0.93–1.26; CC vs. GG: OR = 1.24, 95% CI = 0.91–1.69; CC + GC vs. GG: OR = 1.04, 95% CI = 0.85–1.28; CC vs. GC + GG: OR = 1.23, 95% CI = 0.96–1.57) (see Supplementary Fig. S1). Likewise, no significant association was found in the stratified analysis by ethnicity. In the subgroup analysis by type of leukemia, we found no association between *TP53* Arg72Pro polymorphism and AML in all genetic models. However, we found a significant association between *TP53* Arg72Pro polymorphism and ALL under the recessive model (OR = 1.73, 95% CI = 1.07–2.81, P = 0.025) (Fig. 2). When stratified restricting by control sources, negative results were observed in population-based controls and hospital-based controls.

### Sensitivity analysis and Publication bias

A sensitivity analysis was performed by sequentially omitting individual studies of *TP53* Arg72Pro polymorphism, and the corresponding pooled ORs were not materially changed (see Supplementary Fig. S2). Begg’s funnel plot and Egger’s test were performed to access the publication bias in this meta-analysis. Funnel plot shapes did not reveal any obvious evidence of asymmetry, and the P value of the Egger’s tests was greater than 0.05. The results suggested that publication bias was not evident in this meta-analysis (see Supplementary Fig. S3).

## Discussion

The association between *TP53* genetic mutation and the susceptibility to tumor risk has been confirmed in several functional studies with genetically modified mice, and the results have indicated that mice lacking the inactivating mutation in one *TP53* allele developed fewer tumors than the mice harboring the mutation^25^. Moreover, the *TP53* Arg72Pro polymorphism is the most studied SNP that may be associated with tumor risk. Therefore, it is biologically plausible that this polymorphism has significant association with leukemia progression.

However, previous a meta-analysis has reported that *TP53* Arg72Pro polymorphism was not associated with leukemia risk, even in subgroup analyses by ethnicities and types of leukemia^26^. In the present meta-analysis, with 5 more studies and a larger number of subjects, we reached some meaningful conclusions. The overall summary results of our meta-analysis also suggested no statistically significant risk of *TP53* Arg72Pro polymorphism to leukemia, and no significant association was found in a subgroup analysis by ethnicity. When stratified by types of leukemia, we observed a significant association between *TP53* Arg72Pro polymorphism and ALL risk under the recessive model but not in the AML subjects. In the previous meta-analysis, which was limited by the number of studies, the authors only performed subgroup analyses on the association between *TP53* Arg72Pro polymorphism, the risk of AML (n = 5), and the risk of other types of leukemia (n = 4), and the results were consistent with ours. Thus, our study confirms the association between *TP53* Arg72Pro polymorphism and the risk of leukemia found in previous analysis. However, further studies with larger patient cohorts are needed to clarify this association.

ALL is more common in children, who comprise over 80% of all acute leukemia cases. Childhood ALL shows a consistent association with miscarriages^26^, and the *TP53* Arg72Pro polymorphism has been implicated in implantation failure^27^ and in recurrent miscarriages^28^. However, the association is controversial. Fraga *et al*. reported no association was observed between *TP53* Arg72Pro polymorphism and pregnancy loss risk, but the interaction of the TP53 Arg/Arg (rs1042522) and MDM2 TT (rs2279744) genotypes was shown to increase the risk to pregnancy loss^29^. This feature of childhood ALL makes it an interesting target for the study of TP53 pathway genes. *TP53* Arg72Pro polymorphism may be a modifier of risk for childhood ALL susceptibility and might interact with MDM2. The Arg72 variant is more efficient than the Pro72 variant at inducing apoptosis. The greater apoptotic potential of the Arg72 protein is caused by greater interaction of this protein with MDM2, which facilitates the nuclear export, and probably also the subsequent mitochondrial localization^31^,^32^. Consistent with their biological relationship, our current analysis provides evidence that the *TP53* Arg72Pro polymorphism is associated with an increased risk of developing ALL. To the best of our knowledge, this meta-analysis is the first to investigate the effect of *TP53* Arg72Pro polymorphism on ALL. Furthermore, due to a limited number of available published studies, further studies with larger sample sizes are needed to reach a more convincing conclusion.

In interpreting our results of this meta-analysis, some limitations must be addressed. First, our meta-analysis was based on unadjusted estimates. However, a more precise analysis might be conducted if individual data were available, such an analysis would have allowed us to adjust for other covariates, such as age, family history and environmental factors. Second, the *TP53* Arg72Pro polymorphism might influence susceptibility to leukemia with other factors, but we did not conduct relative research, such as the gene-gene and gene-environment interactions, because the data were insufficient. Third, we could not perform further subgroup stratification analysis by ages because of the limited number of published studies. Finally, publication bias is possible because no attempts were made to identify unpublished studies, although the results of our Begg’s funnel plot and Egger’s test revealed no evidence of publication bias in the allele model.

In conclusion, this meta-analysis did not find any evidence of an association between *TP53* Arg72Pro polymorphism and the risk of leukemia in the overall studies, without the influence of ethnic diversity. However, a statistically significant association was found there was a stronger power trend towards a risk between the *TP53* Arg72Pro polymorphism and ALL. Further studies with larger sample sizes and well-designed studies are required to investigate the associations between *TP53* Arg72Pro polymorphism and leukemia.

## Materials and Methods

### Identification of Eligible Studies

A comprehensive literature search of studies published through December 2014 was performed using the PubMed, EMBASE, Web of Knowledge and the Chinese National Knowledge Infrastructure (CNKI) databases. Various combinations of the terms “TP53 or P53”, “polymorphism or variant”, and “leukemia or leucocythemia” were used to screen for potentially relevant studies. The search was conducted without language restrictions. Additional articles were located manually through the references of the related reports.

### Inclusion and Exclusion Criteria

The following inclusion criteria were applied: a) studies evaluating the association between *TP53* Arg72Pro polymorphism and the risk of leukemia, b) case-control or cohort studies, c) studies with a control genotype distribution in HWE, and d) studies with sufficient genotype data for calculating the odds ratio (OR) with 95% confidence intervals (95% CIs). If studies included overlapping subjects, only the study with a larger sample size was selected. Studies were excluded if one of the following existed: a) not a case-control study, b) no sufficient data were reported, and c) case reports or reviews.

### Data Extraction

Two investigators (Dai and Sun) independently collected the data from each article using a standardized protocol. Disagreement was resolved by discussion with the other co-authors. The following information from the eligible studies was extracted: the first author’s surname, publication year, country of origin, ethnicity, source of control, sample sizes of cases and controls, number of genotypes, *P*-value for Hardy-Weinberg equilibrium (HWE), and genotyping methods. The study design was categorized as a hospital-based study (controls from the hospitalized patients) or a population-based study (controls from the healthy population).

### Quality score assessment

The quality of the studies was independently assessed by the two reviewers (S.J., and J.S) according to the scale for quality assessment (see Supplementary Table S1). These scores were based on both traditional epidemiological considerations and cancer genetic issues. Any disagreement was resolved by discussion between the two reviewers. Total scores ranged from 0 (worst) to 15 (best).

### Statistical analysis

All statistical analyses were performed using Stata software version 12.0 (Stata Corp., College Station, TX, USA), and all *P* values were two-sided. The Hardy-Weinberg equilibrium of controls for each study was tested using the χ^2^ test (significant at the 0.05 level). An OR with the corresponding 95% CI was used to assess the strength of the association between the *TP53* Arg72Pro polymorphism and leukemia susceptibility according to allelic contrast (C vs. G), homozygote comparisons (CC vs. GG), dominant models (CC + GC vs. GG) and recessive models (CC vs. GC + GG). The significance of the summary OR was determined with a Z-test, and a *p* value of less than 0.05 was considered to be statistically significant. Between-study heterogeneity was assessed by calculating the *Q*-statistic and then quantified using the *I*^*2*^ value. When the effects were assumed to be heterogeneous (P < 0.05, I^2^ > 50%), the random-effects model was used; otherwise, the fixed-effects model was more appropriate^33^,^34^. Sensitivity analysis was used to test the stability of pooled studies through sequential omission of individual studies. Potential publication bias was estimated with a Begg’s funnel plot and corroborated with the Egger’s test (P < 0.05 was considered to indicate significant publication bias)^35^,^36^. Additionally, subgroup analyses of ethnicity, source of control and clinical types were performed.

## Additional Information

**How to cite this article**: Tian, X. *et al*. Association between *TP53* Arg72Pro polymorphism and leukemia risk: a meta-analysis of 14 case-control studies. *Sci. Rep.* **6**, 24097; doi: 10.1038/srep24097 (2016).

## Supplementary Material

Supplementary Information

## Figures and Tables

**Table 1:** The characteristics of studies included in the meta-analysis.

First author	Year	Country	Ethnicity	Source of control	Clinical types	Sample size Case/Control	Cases			Controls			HWE	Methods of genotyping	Quality scores
							GG	GC	CC	GG	GC	CC			
Nakano	2000	Japan	Asian	Population based	AML	200/188	82	93	25	59	95	34	0.69	SSCP	13
Bergamaschi	2004	Italy	Caucasian	Population based	CML	96/174	49	37	10	106	61	7	0.63	PCR-RFLP	11
Takeuchi	2005	Japan	Asian	Hospital based	ATL	87/89	33	38	16	32	37	20	0.15	PCR-RFLP	10
Kochethu	2006	UK	Caucasian	Population based	CLL	203/97	119	62	22	44	40	13	0.42	PCR-RFLP	11
Ellis	2008a	USA	Caucasian	Population based	AML	80/2224	42	35	3	1255	838	131	0.57	Taqman	15
Ellis	2008b	UK	Caucasian	Population based	AML	91/798	53	31	7	459	289	50	0.62	PCR-RFLP	14
Phang	2008	Singapore	Asian	Population based	MIX	44/160	13	25	6	56	72	32	0.32	PCR-RFLP	11
Xiong	2009	China	Asian	Hospital based	AML	231/128	52	127	52	39	64	25	0.89	PCR-RFLP	12
Do	2009	US	Caucasian	Population based	ALL	114/414	50	45	19	234	154	26	0.92	Taqman	13
Shi	2011	China	Asian	Population based	AML	180/555	55	84	41	171	289	95	0.15	MALDI-TOF	13
Chauhan	2012	India	Asian	Population based	AML	131/199	38	71	22	51	112	36	0.06	PCR-RFLP	12
					ALL	99/199	28	43	28	51	112	36	0.06	PCR-RFLP	12
Dunna	2012	India	Asian	Population based	ALL	147/245	59	67	21	79	123	43	0.68	PCR-RFLP	12
					AML	141/245	64	44	33	79	123	43	0.68	PCR-RFLP	12
Chen	2013	China	Asian	Population based	ALL	174/356	39	90	45	113	183	60	0.33	PCR-RFLP	13
Perim	2013	Brazil	Mixed	Population based	ALL	54/58	24	23	7	37	19	2	0.82	PCR-RFLP	10

*HWE*, Hardy-Weinberg equilibrium; *PCR*, polymerase chain reaction; *RFLP*, restriction fragment length polymorphism; *SSCP*, Single-Strand Conformation Polymorphism.

ALL, Acute lymphoblastic leukemia; AML, Acute myeloid leukemia; CLL, Chronic lymphocytic Leukemia; CML, Chronic myeloid leukemia; ATL, Adult T-cell leukemia.

**Table 2:** Meta-analysis of the association between *TP53* Arg72Pro polymorphism and leukemia.

Subgroup	NO.	C vs. G			CC vs. GG			CC + GC vs. GG			CC vs. GC + GG		
		OR(95% CI)	P_h_	P_OR_	OR (95% CI)	P_h_	P_OR_	OR(95% CI)	P_h_	P_OR_	OR(95% CI)	P_h_	P_OR_
Overall	14	1.09(0.93–1.26)	0.000	0.272	1.24(0.91–1.69)	0.001	0.203	1.04(0.85–1.28)	0.001	0.530	1.23(0.96–1.57)	0.014	0.106
Ethnicity													
Asian	8	1.02(0.86–1.20)	0.012	0.847	1.07(0.77–1.49)	0.024	0.678	0.98(0.76–1.27)	0.011	0.894	1.16(0.96–1.38)	0.130*	0.120*
Caucasian	5	1.15(0.83–1.59)	0.002	0.415	1.44(0.68–3.04)	0.005	0.337	1.11(0.78–1.59)	0.018	0.552	1.44(0.76–2.72)	0.022	0.263
Mixed	1	2.11(1.15–3.86)	–	0.016	5.40(1.03–28.19)	–	0.046	2.20(1.03–4.70)	–	0.041	4.17(0.83–21.04)	–	0.084
Source of control													
Population-based	13	0.88(0.74–1.04)	0.000	0.127	0.78(0.55–1.10)	0.001	0.158	0.74(0.56–0.98)	0.005	0.038	0.93(0.75–1.16)	0.004	0.529
Hospital-based	2	0.90(0.71–1.16)	0.197*	0.423	0.83(0.51–1.37)	0.185*	0.469	0.97(0.63–1.49)	0.353*	0.885	0.81(0.55–1.18)	0.214*	0.267
Clinical types													
AML	7	0.98(0.88–1.10)	0.179*	0.730*	1.00(0.79–1.27)	0.208*	0.986*	0.91(0.72–1.16)	0.048	0.448	1.11(0.89–1.37)	0.277*	0.351*
ALL	5	1.33(0.97–1.82)	0.002	0.078	1.83(0.95–3.50)	0.003	0.069	1.26(0.84–1.90)	0.008	0.266	1.73(1.07–2.81)	0.022	**0.025**
Others#	4	0.97(0.68–1.39)	0.035	0.882	0.95(0.61–1.48)	0.087*	0.827*	0.97(0.73–1.28)	0.056*	0.823*	0.93(0.62–1.40)	0.129	0.722*

*OR* odds ratio; 95% *CI*, 95% confidence interval; *P*_*OR*_, pool *P* value; *P*_*h*_, *P* value of heterogeneity test; NA, data not available.

^*^Estimates for fixed-effects model; otherwise, random-effects model was used.

^#^Others include chronic lymphocytic leukemia, chronic myeloid leukemia, adult T-cell leukemia, and mixed types.
