# Supplementary material for: Association between TP53 Arg72Pro polymorphism and leukemia risk: a meta-analysis of 14 case-control studies
Source: Sci Rep. 2016 Apr 7;6:24097. doi: 10.1038/srep24097 (PMC4823650; doi:10.1038/srep24097)

Association between *TP53* Arg72Pro polymorphism and leukemia risk: a meta-analysis of 14 case-control studies

Xin Tian[1]*, Shundong Dai[2,3], Jing Sun[4], Shenyi Jiang[5], Youhong Jiang[1]

1. Molecular Oncology Laboratory of Cancer Research Institute, the First Affiliated Hospital of China Medical University, Shenyang, 110001, PR China
2. Department of Pathology, the First Affiliated Hospital and College of Basic Medical Sciences of China Medical University, Shenyang, 110001, PR China
3. Institute of Pathology and Pathophysiology, Shenyang, 110001, PR China
4. Department of Immunology and Biotherapy, Liaoning Cancer Hospital and Institute, Shenyang, 110042, PR China
5. Department of Rheumatology, the First Affiliated Hospital of China Medical University, Shenyang, 110001, PR China

* Corresponding author: Xin Tian

Molecular Oncology Laboratory of Cancer Research Institute, the First Affiliated Hospital of China Medical University, Shenyang 110001, PR China.

E-mail address: tianxin@cmu1h.com

Tel.: +86 24 83282354

Fax: +86 24 83282473

Table S1 Scale for quality assessment criterion

Criterion	Score
**Source of cases**	
Selected from population or cancer registry	3
Selected from hospital	2
Selected from pathology archives, but without description	1
Not described	0
**Source of controls**	
Population-based	3
Blood donors or volunteers	2
Hospital-based (cancer-free patients)	1
Not described	0
**Specimens used for determining genotypes**	
White blood cells or normal tissues	3
Tumor tissues or exfoliated cells of tissue	0
**Hardy–Weinberg equilibrium in controls**	
Hardy–Weinberg equilibrium	3
Hardy–Weinberg disequilibrium	0
**Total sample size**	
>1,000	3
>500 and <1,000	2
>200 and <500	1
<200	0

Fig.S1 Forest plot of OR with 95% CI of leukemia associated with the *TP53* Arg72Pro polymorphism (a allele model; b homozygous model; c dominant model; d recessive model)

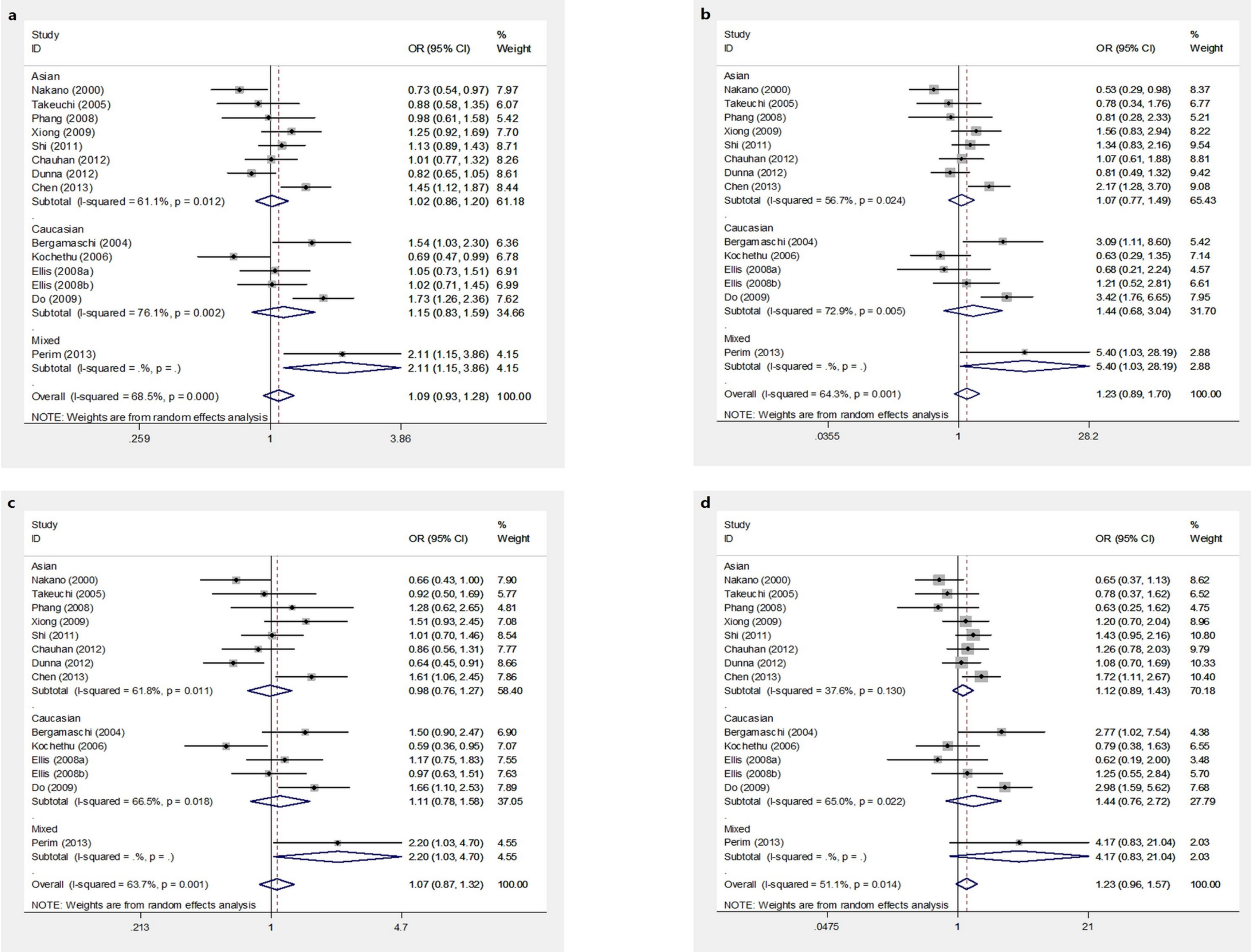

Fig.S2 Sensitivity analysis of the association between the *TP53* Arg72Pro polymorphism and leukemia (allele model)

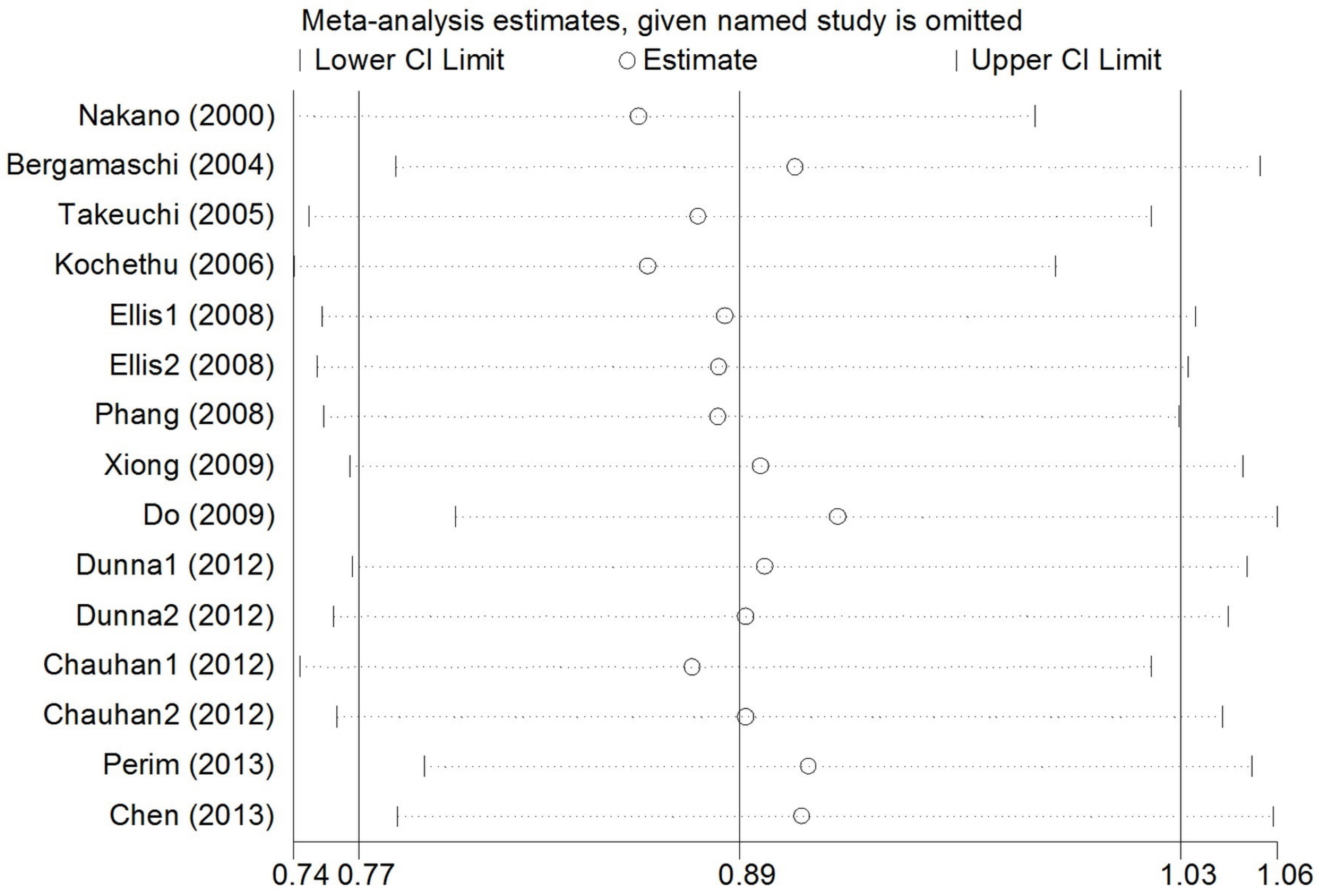

Fig.S3 Begger's funnel plot of publication biases on the association between the *TP53* Arg72Pro polymorphism and leukemia risk (allele model)

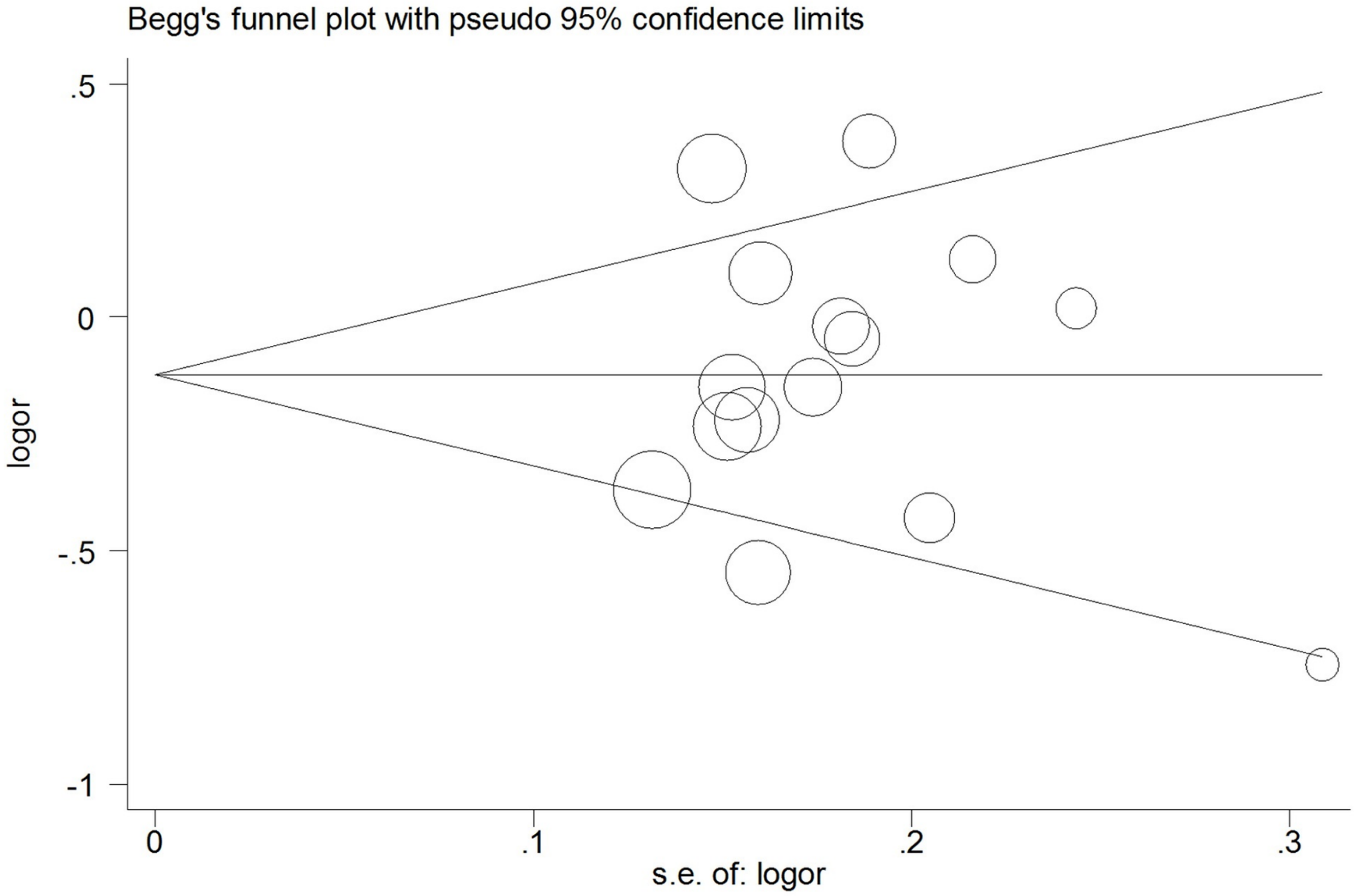

Supplement: Supplementary Information [file srep24097-s1.pdf]
